# Economic, ethical, and regulatory dimensions of artificial intelligence in healthcare: an integrative review

**DOI:** 10.3389/fpubh.2025.1617138

**Published:** 2025-08-29

**Authors:** Rabie Adel El Arab, Omayma Abdulaziz Al Moosa, Mette Sagbakken

**Affiliations:** ^1^Almoosa College of Health Sciences, Alhsa, Saudi Arabia; ^2^Department of Nursing and Health Promotion, Faculty of Health Sciences, Oslo Metropolitan University, Oslo, Norway

**Keywords:** artificial intelligence, economic evaluation, ethical oversight, regulatory harmonization, governance frameworks, risk management, clinical implementation, reimbursement models

## Abstract

**Background:**

Artificial Intelligence (AI) is revolutionizing healthcare by improving diagnostic precision, streamlining clinical workflows, and reducing operational costs. Yet, its integration into real-world settings remains fraught with challenges—including economic uncertainty, ethical complexities, fragmented regulatory landscapes, and practical implementation barriers. A growing body of literature highlights that many of AI’s purported benefits are derived from idealized models, often failing to reflect the nuances of clinical practice.

**Objectives:**

This integrative review aims to critically evaluate the current evidence on the integration of artificial intelligence into healthcare, with a particular focus on its economic impact, ethical and regulatory challenges, and associated governance and implementation strategies.

**Methods:**

A comprehensive literature search was conducted across PubMed/MEDLINE, Embase, Web of Science, and the Cochrane Library. Data extraction followed a structured, pre-tested template, and thematic synthesis was employed. Study quality was assessed using an integrated framework combining PRISMA, AMSTAR 2, and the Drummond checklist.

**Results:**

Seventeen studies—including systematic reviews, scoping reviews, narrative syntheses, policy analyses, and quantitative case studies—met the inclusion criteria. Three core themes emerged from the analysis. First, while AI interventions—particularly in treatment optimization—are projected to generate significant cost savings and improve operational efficiency, most economic evaluations rely on theoretical models. Many lack transparency regarding key assumptions such as discount rates, sensitivity analyses, and real-world implementation costs, limiting their generalizability. Second, ethical and regulatory concerns persist, with widespread underrepresentation of marginalized populations in training datasets, limited safeguards for patient autonomy, and notable equity disparities across clinical domains. Regulatory frameworks remain fragmented globally, with marked variation in standards for cybersecurity, accountability, and innovation readiness. Third, effective governance and risk management are critical for ensuring safe and sustainable AI integration. Persistent implementation barriers—such as clinician trust deficits, cognitive overload, and data interoperability challenges—underscore the need for robust multidisciplinary collaboration.

**Recommendations:**

To address these challenges, we present the IA^2^TF Framework—a theoretical model pending empirical validation. It is built on five pillars: co-design and problem definition, data standardization, real-world performance monitoring, ethical and regulatory integration, and multidisciplinary governance. This framework offers an actionable roadmap for fostering equitable, trustworthy, and scalable AI deployment across healthcare systems.

**Conclusion:**

Maximizing the transformative potential of AI in healthcare will require rigorous economic evaluation, equity-driven design, harmonized global regulation, and inclusive implementation science. The IA^2^TF Framework provides a foundation for ethically grounded, patient-centered, and financially sustainable AI integration.

## Introduction

Artificial Intelligence (AI) is rapidly transforming the landscape of healthcare, promising substantial enhancements in patient care, operational efficiency, and overall system sustainability (1–3). Recent advancements in machine learning (ML), deep learning (DL), and large language models (LLMs) have propelled the development of sophisticated AI applications capable of significantly improving diagnostic accuracy, personalizing treatment plans, and optimizing clinical workflows across diverse medical disciplines, including radiology, oncology, cardiology, and primary care (4, 5). For instance, DL algorithms in medical imaging have shown diagnostic performance comparable to that of expert clinicians, and AI-driven prognostic models in oncology support earlier, more precise interventions (6–8). However, these promising results are often derived from studies with heterogeneous designs and idealized assumptions—such as hypothetical cost models or retrospective validations—which may not fully reflect real-world clinical complexity (9). As such, these findings should be interpreted cautiously, particularly when extrapolated to broader healthcare systems.

These advancements position AI as a crucial element in addressing escalating pressures faced by global healthcare systems, including rising healthcare expenditures driven by aging populations, increased chronic disease prevalence, and complex healthcare needs (10–12). It is therefore essential to interpret these benefits within the context of methodological variability and real-world challenges (13).

Despite these promising benefits, AI integration into healthcare also introduces complex challenges across economic, ethical, regulatory, and practical implementation domains (14, 15). Recent reviews highlights risks such as unpredictable algorithmic errors, insufficient regulatory oversight, rising implementation costs, and equity concerns—issues that may compromise patient safety and trust if not proactively addressed (16, 17).

Economically, while AI offers compelling potential to reduce healthcare costs through improved diagnostic accuracy and operational efficiency, existing economic evaluations of AI interventions often exhibit methodological inconsistencies and fragmented reimbursement strategies, complicating the determination of AI’s true financial value and sustainability (18, 19). Ethical concerns related to AI include significant critical issues such as algorithmic bias, fairness, patient autonomy, and data privacy, with evidence indicating that biases in training datasets can exacerbate healthcare disparities, particularly affecting marginalized populations (20, 21). It is essential to acknowledge that while proposals such as the FAIR statement are promising, they may not fully address all ethical challenges inherent to AI integration (22). Additionally, the complexity and opacity of deep-learning models present transparency and accountability challenges, underscoring the need for robust ethical oversight mechanisms (23, 24).

The global regulatory landscape further complicates AI integration, characterized by significant variation in frameworks across jurisdictions (25, 26). We caution that the challenges of harmonizing these diverse regulatory approaches are profound and may be underestimated in some analyses. Regulatory fragmentation poses barriers to global standardization, affecting patient safety, ethical oversight, and international collaboration. Practical challenges, including limited interoperability, inadequate governance structures, clinician resistance, and performance decline when AI models transition from controlled environments to real-world clinical settings, further complicate the effective and sustainable implementation of AI (27–29). Although previous reviews have addressed AI applications in specific domains—such as emergency department triage, nursing education and practice, and vaccine research and development—none provides an integrative synthesis that simultaneously examines the economic and regulatory dimensions of AI across diverse clinical contexts. In particular, key considerations such as cost-effectiveness evaluations, reimbursement frameworks, and real-world implementation feasibility are either absent or insufficiently explored in the existing literature (30–32).

This fragmented evidence base highlights a significant knowledge gap: the absence of a comprehensive assessment that synthesizes AI’s multifaceted impacts while adequately evaluating the quality and methodological rigor of the available studies. Addressing this gap is essential for the responsible advancement of healthcare AI, requiring consolidated evidence that thoroughly assesses AI’s economic viability, ethical integrity, regulatory coherence, and practical governance strategies.

### Aim

This integrative review aims to critically assess current evidence on the integration of AI into healthcare, with a particular emphasis on its economic impact, ethical and regulatory challenges, and governance and implementation strategies.

### Objectives

1. Assess how AI contributes to cost-effectiveness and scalability in healthcare, including an assessment of the robustness of economic evaluations and the efficiency of reimbursement strategies.
2. Examine the ethical implications of AI in healthcare by exploring issues of fairness, bias mitigation, and patient autonomy, and evaluate the performance of various global regulatory frameworks.
3. Investigate the role of governance structures and risk management practices in AI integration and identify key enablers and barriers influencing its safe and sustainable adoption in clinical practice.
4. To propose a comprehensive framework for the integration of AI in healthcare that combines best practices and addresses identified gaps in economic evaluation, ethical oversight, regulatory governance, and practical implementation.

## Methods

This integrative review was undertaken to synthesize evidence from a heterogeneous body of literature, including systematic reviews, scoping reviews, narrative reviews, policy analyses, validation studies, and quantitative case studies, addressing the multifaceted applications, challenges, and impacts of AI in healthcare. Given the diversity of study designs, we have made a concerted effort to differentiate the quality and generalizability of findings across the various methodologies. This systematic review was conducted in accordance with the Preferred Reporting Items for Systematic Reviews and Meta-Analyses (PRISMA) 2020 statement (33). To ensure the relevance and quality of the included studies, specific inclusion and exclusion criteria were established (Table 1).

**Table 1:** Inclusion and exclusion criteria.

Criterion	Inclusion	Exclusion
Population	Studies conducted in clinical or health policy settings where AI is applied to patient care or system-wide decision-making.	Studies focused exclusively on non-clinical settings or on the technical development of AI algorithms without clinical implementation.
Intervention	Use of AI technologies (e.g., machine learning, deep learning, LLMs), integrated into clinical workflows, decision support, or health policy development.	Studies reporting solely on algorithm performance without addressing clinical, economic, or governance implications.
Outcomes	Reporting of evaluative endpoints including economic metrics, fairness assessments, risk management outcomes, regulatory and governance insights.	Studies without clear evaluative or outcome data, or those not contextualizing findings within clinical or policy frameworks.
Study design	Peer-reviewed primary research (RCTs, observational studies, economic analyses, validation studies, case studies) and systematic/scoping/narrative reviews.	Editorials, commentaries, abstracts without full-text data, or studies lacking detailed methodological descriptions.
Language	Publications in English.	Non-English publications.

### Inclusion and exclusion criteria

Stringent eligibility criteria were established *a priori* to ensure both clinical relevance and methodological rigor. Table 1 details the specific inclusion and exclusion criteria applied during study selection.

### Literature search and study selection

A comprehensive literature search was performed across the following electronic databases: PubMed/MEDLINE, Embase, Web of Science, and the Cochrane Library. The search aimed to capture all relevant articles published from the inception of each database inception up to October 2024, with additional research conducted in March 2025 to identify newly published articles. The search strategy combined both controlled vocabulary (e.g., MeSH, Emtree) and free-text terms across three core domains: AI technologies (e.g., “artificial intelligence,” “machine learning,” “deep learning,” “LLMs”), clinical applications (e.g., “radiology,” “health policy,” “clinical decision support”), and evaluative or contextual outcomes (e.g., “economic evaluation,” “risk management,” “fairness,” “governance,” “regulatory,” “implementation”). Table 2 summarizes the database-specific search strings.

**Table 2:** Search strategy by database.

Database	Search string
PubMed/MEDLINE	(“Artificial Intelligence”[MeSH] OR “machine learning” OR “deep learning” OR “LLMs”) AND (“Cost Effectiveness”[MeSH] OR “economic evaluation” OR “fairness” OR “governance” OR “risk management” OR “regulatory”) AND (clinical OR healthcare OR “health policy”)
Embase	(‘artificial intelligence’/exp. OR “machine learning” OR “deep learning” OR “LLMs”) AND (“cost effectiveness” OR “economic evaluation” OR “fairness” OR “governance” OR “risk management” OR “regulatory”) AND (clinical OR healthcare)
Web of science	TS = (“artificial intelligence” OR “machine learning” OR “deep learning” OR “LLMs”) AND TS = (“economic evaluation” OR “fairness” OR “governance” OR “risk management” OR “regulatory”) AND TS = (clinical OR healthcare OR “health policy”)
Cochrane library	(“Artificial Intelligence” OR “machine learning” OR “deep learning” OR “LLMs”) in Title, Abstract, Keywords AND (“economic evaluation” OR “fairness” OR “governance” OR “risk management” OR “regulatory”) in Title, Abstract, Keywords

### Data extraction and quality appraisal

**Figure 1:**
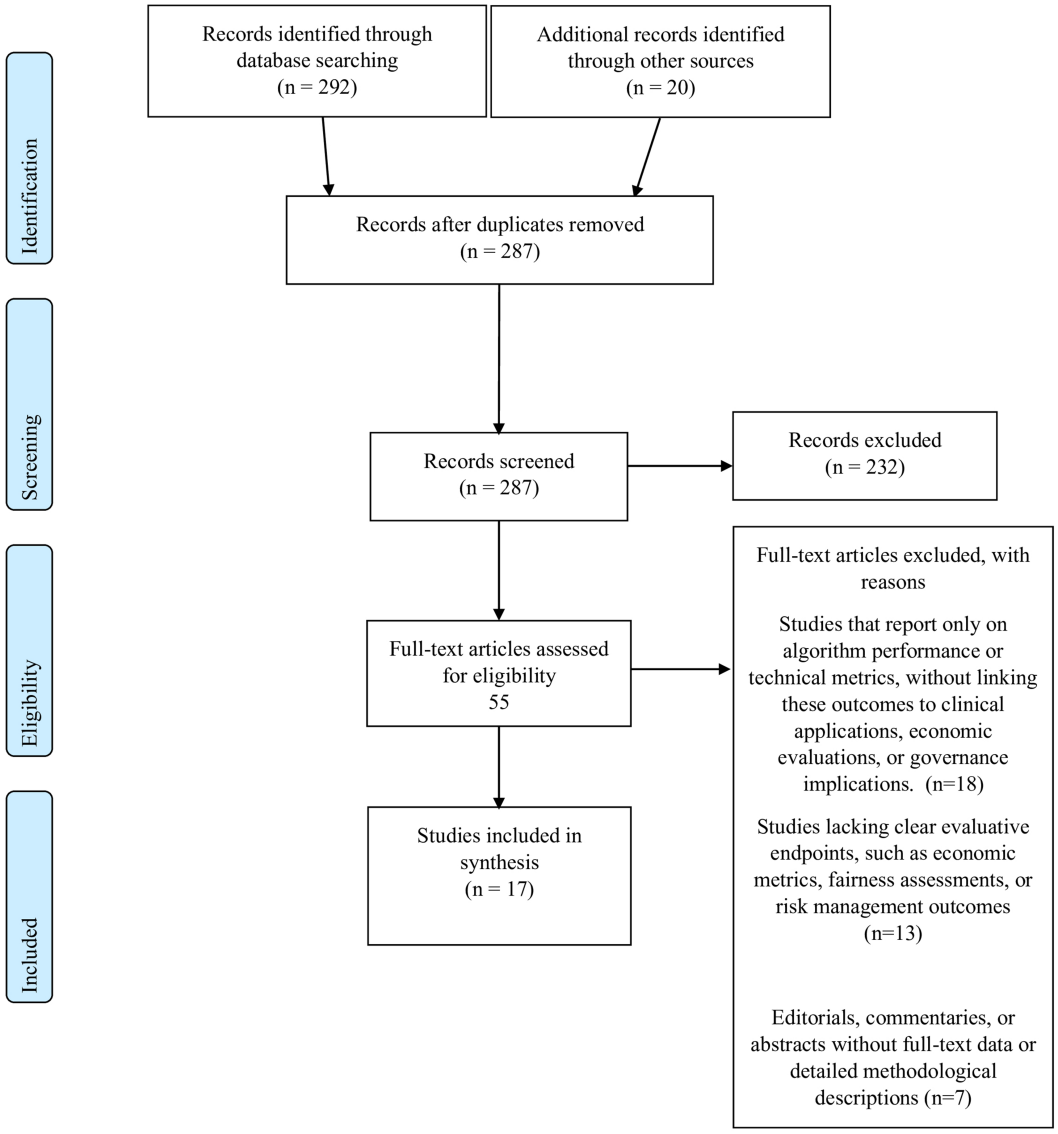
PRISMA flow diagram for study selection.

All records retrieved from the database searches were imported into a dedicated systematic review management platform Rayyan (34). Two independent reviewers screened titles and abstracts against the eligibility criteria. Full texts of potentially eligible articles were then retrieved and assessed independently; discrepancies were resolved by consensus. Data were extracted using a pre-piloted standardized form that captured essential details, such as study identification (authors and year), study design and methodology, study objectives and settings, key methods and frameworks employed, and the main findings, recommendations, and limitations. The entire study selection process, including detailed reasons for exclusion at the full-text stage, is documented in the PRISMA 2020 flow diagram provided in Figure 1.

### Data synthesis

Data synthesis was undertaken using a thematic synthesis approach guided by established methodologies (35–37). After data extraction, two reviewers independently mapped each study’s key findings onto the three pre-specified analytic domains: (1) economic and financial implications; (2) ethical, regulatory and equity dimensions; and (3) governance, risk management and implementation science—using a tabular framework. Within each domain we summarized and compared outcome measures, noted areas of convergence and divergence, and identified cross-cutting issues. Disagreements in mapping or interpretation were resolved through discussion with a third reviewer. Because of substantial clinical and methodological heterogeneity across the included studies, statistical pooling was inappropriate; therefore, results are presented as a narrative synthesis.

### Composite quality assessment

In this integrative review, a composite quality assessment strategy was adopted to evaluate the diverse studies included, ranging from systematic reviews and empirical economic evaluations to narrative commentaries and innovative framework proposals. This approach was chosen because it not only assesses the risk of bias—a critical component in evaluating internal validity—but also incorporates broader quality indicators such as clarity, methodological transparency, and the appropriateness of study design.

The heterogeneity of the studies under review necessitated a strategy that could address multiple methodological approaches. Traditional risk-of-bias tools, such as those described in Higgins et al. (38), are optimized for homogeneous study designs and often do not translate well to reviews that include non-randomized or qualitative designs. Therefore, integrating broader quality appraisal criteria ensures that key aspects—including reporting transparency, theoretical grounding, and methodological rigor—are adequately captured. Therefore, our composite quality assessment framework explicitly differentiates between studies with rigorous risk-of-bias evaluations and those with less formal methodological scrutiny, ensuring that weaker evidence does not unduly influence overall conclusions. While risk of bias focuses on internal validity by identifying potential systematic errors that might affect study outcomes, overall quality appraisal extends this evaluation to include external validity and methodological clarity. For example, systematic reviews in our sample were evaluated using components derived from the PRISMA guidelines (33) and AMSTAR 2 (39), whereas economic evaluations were appraised with instruments such as the Drummond checklist (40). This stratified approach ensures that findings from highly rigorous studies are distinguished from those based on less robust methodologies. Combining these elements allows for a comprehensive assessment that reflects both the rigor and the practical applicability of the findings.

In the context of healthcare—and particularly for emerging technologies such as artificial intelligence—it is crucial that the evaluation framework addresses not only methodological soundness but also the relevance and applicability of the study findings to clinical and policy decision-making. Reviews that incorporate frameworks such as the Consolidated Framework for Implementation Research (CFIR) (41).

To integrate evidence from diverse study designs, we adopted a two-phase composite quality assessment approach. In phase one, we extracted quality indicators using the established tools PRISMA (33), AMSTAR 2 (39), and the Drummond checklist (40). In phase two, these indicators were used to assign weights to studies—granting higher weight to robust systematic reviews and empirical evaluations—thereby ensuring that less rigorous evidence was proportionately down-rated.

This included evaluating internal validity through factors such as study design, sample selection, and potential confounders; assessing methodological rigor by examining the transparency of the search strategy, data collection methods, and analytical approaches; and considering reporting clarity and relevance by evaluating how well the study’s objectives, limitations, and implications were described. This comprehensive framework enabled a balanced appraisal of each study, ensuring that studies with robust internal validity but limited external applicability, or vice versa, were appropriately weighted in our synthesis.

By integrating both risk-of-bias assessment and broader quality appraisal measures, our composite framework provides a balanced and comprehensive evaluation of each study’s methodological quality. We stress that, due to inherent heterogeneity, conclusions drawn must be tempered by an understanding of the underlying study limitations, particularly where methodological rigor is variable. This approach is especially well-suited for the heterogeneous body of literature in AI healthcare research, ensuring that our synthesis is both rigorous and practically relevant for informing clinical practice and policy (Appendix 2). The study selection process (see Figure 1) was instrumental in ensuring that only studies meeting rigorous inclusion criteria were considered. This process directly informed our composite quality assessment, where studies were weighted based on their methodological soundness and reporting clarity. By linking the selection criteria to the quality evaluation, we aimed to mitigate the risk of overgeneralizing findings from studies with inherent methodological limitations.

## Results

Our synthesis encompasses 17 studies published between 2021 and 2025, representing a broad spectrum of research designs and healthcare domains—including radiology, health policy, reimbursement strategies, clinical risk management, and personalized care (9, 42–57). The studies span diverse settings from specialized hospital departments and global regulatory contexts to traditional firms, and employ varied methodologies such as scoping reviews, narrative and policy analyses, prospective validation studies, and quantitative case studies. Key frameworks and methods used include the FAIR recommendations, policy triangle, CFIR framework, economic modeling, ERM, HEAL, and network theory, with evaluation metrics ranging from diagnostic performance and cost-effectiveness to equity assessment and risk mapping (Appendix 1).

### Thematic synthesis

The Results section has been streamlined into three themes: (1) Economic and Financial Implications, (2) Ethical, Regulatory, and Equity Dimensions, and (3) Governance, Risk Management, and Implementation Science.

#### Theme 1: economic and financial implications

##### Subtheme 1.1: cost-effectiveness, scalability, and reimbursement

Khanna et al. (53) modeled the economic impact of AI adoption across 20 hospitals treating 20 patients daily over a 10-year horizon. Their analysis estimates that AI-enabled treatment applications could yield cumulative savings exceeding $2.3 million per institution, significantly outperforming diagnostic AI applications, which demonstrated approximately 40% lower savings. Additionally, AI systems substantially reduced diagnostic time—from 3.3 h daily in Year 1 to 15.2 h daily by Year 10—contributing to reduced labor costs and improved clinical throughput. These projections underscore the strategic financial advantage of deploying AI in treatment contexts for maximizing economic efficiency and operational scalability.

Complementing these findings, Moro-Visconti et al. (44) support these projections with sensitivity models showing that a 5–10% increase in revenue or equivalent cost reductions through AI integration can substantially enhance financial performance, potentially doubling or tripling firm valuation. Their analysis emphasizes AI’s contribution to financial sustainability by framing it not as a sunk cost, but as a scalable, revenue-generating asset. While the authors do not explicitly use the term “real-options valuation,” their approach highlights the strategic and dynamic value of AI investments in reshaping traditional business models and boosting enterprise resilience. Reimbursement remains a pivotal enabler of AI scalability. Abramoff et al. (56), demonstrate that Value-Based Care (VBC) models can offer up to 10 times higher reimbursement rates for autonomous AI solutions (e.g., diabetic eye screening) compared to Fee-for-Service (FFS) models. Their case illustrates that these elevated rates are contingent upon achieving population health metrics such as HEDIS, MIPS, or RAF scores. While exact reimbursement figures are not disclosed, the analysis underscores the financial imperative for aligning AI implementation with performance-based reimbursement frameworks. The authors advocate for hybrid reimbursement models that incentivize innovation while maintaining fiscal sustainability.

##### Subtheme 1.2: methodological rigor in economic evaluations

Despite promising projections, both Kastrup et al. (43) and Khanna et al. (53) highlight that many economic evaluations of AI in healthcare suffer from methodological limitations. For example, Kastrup et al. found that although 62% of studies explicitly reported Incremental Cost-Effectiveness Ratios (ICERs), many lacked justification for the evaluation methods used or did not fully disclose modeling assumptions such as discount rates, sensitivity analyses, or dynamic pricing mechanisms.

Common omissions include real-world implementation costs (e.g., staff training, IT integration delays) and limited stratification by clinical condition, which restricts generalizability. Khanna et al. emphasized that domains like oncology and cardiology demonstrate the highest cost-saving potential—often in the range of hundreds of dollars per patient—particularly for risk stratification tasks. However, areas such as psychiatry and primary care remain underrepresented, with sparse or missing economic data.

Although economic analyses suggest substantial cost reductions from AI interventions, these models are largely hypothetical and often assume ideal implementation conditions. Evidence from a prospective validation study in our sample illustrates this limitation: the VinDr-CXR system’s F1 score declined from 0.831 during development to 0.653 in real-world clinical practice (55). This notable performance drop highlights the uncertainty surrounding projected economic savings and emphasizes the need for cautious interpretation of modeled benefits.

#### Theme 2: ethical, regulatory, and equity dimensions

##### Subtheme 2.1: fairness, Bias, and ethical challenges

Ueda et al. (9) emphasize that minority underrepresentation in AI training datasets can lead to significant algorithmic bias, potentially exacerbating diagnostic disparities across ethnic subgroups. While the review does not report specific sensitivity differentials, it highlights this issue as a key source of inequity in AI-driven healthcare systems. In parallel, Mennella et al. (49) underscore the ethical imperative of maintaining patient autonomy and ensuring informed consent in AI-assisted clinical decisions. They argue that robust ethical oversight is necessary to prevent AI from perpetuating existing healthcare inequities, thereby emphasizing the need for transparent and accountable AI systems that support rather than supplant human judgment. The HEAL framework (48) quantified equity prioritization at 92.1% for sex-based subgroups, demonstrating strong AI performance toward females with poorer health outcomes. In contrast, the framework yielded a HEAL score of 0.0% for older adults in non-cancer dermatologic conditions, underscoring domain-specific inequities and the need for targeted improvements.

##### Subtheme 2.2: global regulatory frameworks and digital innovation in personalized healthcare

The regulatory landscape for AI in healthcare is evolving rapidly across different regions. Pesapane et al. (47) provide a comparative analysis that illustrates how the European Union, the United States, China, and Russia are each addressing challenges related to data security, accountability, and cybersecurity. European frameworks, bolstered by the Medical Device Regulation and GDPR, contrast with the adaptive approaches seen in the United States—such as the FDA’s Software as a Medical Device categorization—and with emerging guidelines in China and Russia. Simultaneously, Li et al. (45) and Wang and Zhang (46) delve into the digital innovations that are transforming personalized healthcare. They examine the potential of virtual assistant chatbots, remote patient monitoring, predictive analytics, and LLM to revolutionize clinical practice, while also identifying challenges related to data interoperability and inherent algorithmic biases. This dual focus on regulatory evolution and digital innovation highlights the complex interplay between ensuring safety and fostering technological advancement.

#### Theme 3: governance, risk management, and implementation science

##### Subtheme 3.1: governance structures and risk mitigation strategies

Effective governance and proactive risk management are essential for the safe integration of AI in clinical practice. Liao et al. (54) detail a comprehensive governance framework established within a large health system, organized into clinical, operational, and leadership domains. This framework has enabled systematic performance monitoring, guided strategic decisions about AI deployment and retirement, and reinforced accountability. In parallel, Nguyen et al. (55) report on the prospective validation of the VinDr-CXR system, observing a notable decline in performance when transitioning from laboratory settings to real-world application. Their findings—along with those of Di Palma et al. (50) who advocate for the adoption of Enterprise Risk Management methodologies such as Failure Mode, Effects, and Criticality Analysis—emphasize the need for continuous local retraining and detailed risk mapping to ensure patient safety.

##### Subtheme 3.2: implementation barriers, facilitators, and multidisciplinary collaboration

The successful implementation of AI in healthcare hinges on overcoming substantial barriers and leveraging critical facilitators. Chomutare et al. (51) synthesize evidence from a range of studies to identify key enablers, including robust leadership support, active clinician engagement, and stringent validation processes. They also pinpoint barriers such as interoperability challenges, data quality issues, and prevailing trust deficits that can impede adoption. Further enriching this discussion, Ferrara et al. (52) and Darwiesh et al. (42) illustrate that even as AI systems improve diagnostic support and operational efficiency, they may also introduce new risks—such as cognitive overload among clinicians and emerging vulnerabilities detected via social media analytics. These insights collectively demonstrate that multidisciplinary collaboration is indispensable for addressing technical, clinical, and operational challenges and for ensuring the safe, effective, and sustainable implementation of AI.

### Composite quality assessment

Our composite quality assessment included 17 studies, which varied widely in design—from systematic reviews and empirical economic analyses to narrative commentaries and innovative model proposals. Given the diversity in study design and methodological quality, we have explicitly weighed the contributions of each study according to its rigor.

The assessment revealed that narrative reviews, such as those by Ueda et al. (9), Mennella et al. (49), and Li et al. (45), provided comprehensive discussions of the topic and valuable theoretical insights, but generally lacked structured risk-of-bias assessments. These methodological limitations are explicitly acknowledged and have been factored into the synthesis to avoid overgeneralization of their conclusions. In contrast, systematic reviews by Ferrara et al. (52) and Kastrup et al. (43) exhibited high methodological rigor with detailed search strategies, comprehensive data synthesis, and formal risk-of-bias evaluations. Empirical economic analyses, including those by Khanna et al. (53) and Moro-Visconti et al. (44), applied robust economic modeling techniques and sensitivity analyses, underscoring the potential of integrating economic evaluations into the broader AI healthcare literature. However, we caution that even these economic models rely on assumptions that may overestimate benefits when applied to diverse real-world settings.

Frameworks such as the HEAL model in Schaekermann et al. (48) and the integrated enterprise risk management approach described by Di Palma et al. (50) contributed quantitative methodologies to the field. These studies effectively bridged methodological rigor with practical insights, offering actionable recommendations for performance equity and risk management in clinical settings. Moreover, cross-national regulatory reviews, exemplified by Pesapane et al. (47), provided a thorough comparative analysis of legal frameworks and policy challenges, particularly in the post-pandemic context. Nonetheless, we underline that regulatory challenges remain more complex than these comparative analyses might suggest, particularly when considering global standardization.

Overall, while the studies vary in methodological approach and depth of bias assessment, the composite quality assessment framework allowed us to capture both the strengths and limitations of each study. This heterogeneity is clearly acknowledged throughout the synthesis, and the resulting conclusions are presented with appropriate caution regarding their generalizability. This heterogeneity underscores the importance of using a balanced synthesis approach in our integrative review. The insights derived from the quality assessment serve as a foundation for interpreting the overall evidence, ensuring that conclusions drawn from the literature are both robust and practically relevant.

## Discussion

The objectives of this review were to evaluate the economic impact, ethical challenges, and governance frameworks associated with AI integration in healthcare.

Our findings, organized into three core themes, directly address these objectives. Specifically, the economic analyses underscore both the potential cost-savings and the limitations inherent in simulated models; the ethical reviews reveal significant concerns regarding bias and patient autonomy; and the governance discussions highlight the need for robust, adaptive frameworks to support real-world AI implementation. This structured approach ensures that our discussion, recommendations, and conclusions are firmly rooted in the initial aims of the study.

The integration of AI into healthcare signifies a transformative shift with profound implications for patient care, operational efficiency, and the overall medical landscape. While AI offers remarkable potential, its deployment raises critical considerations regarding economic sustainability, ethical standards, regulatory frameworks, and governance mechanisms. This discussion delves into these multifaceted issues, interpreting our review’s findings and juxtaposing them with existing literature to elucidate the path toward responsible and effective AI integration in healthcare. Across the 17 included studies, several recurrent methodological gaps emerged. Most economic evaluations relied on hypothetical or retrospective models and provided only limited, non-granular accounting of real-world cost components—such as IT integration, clinician training, and workflow redesign—thereby constraining practical applicability. Reporting standards and quality-appraisal frameworks varied widely, with sensitivity analyses, discount-rate assumptions, and formal risk-of-bias assessments seldom reported. External validity was further limited by small, single-center cohorts or narrow clinical domains, and prospective validations remain rare. These weaknesses underscore the need for standardized reporting guidelines and real-world pilot studies to strengthen the evidence base.

Our review underscores AI’s potential to enhance diagnostic accuracy and streamline clinical processes, leading to significant cost reductions. However, we caution that many economic advantages stem from models based on assumptions that may not apply universally; therefore, these benefits should be considered context-dependent rather than universally assured. This aligns with existing literature emphasizing AI’s role in improving healthcare service quality and operational efficiency. For instance, studies have highlighted AI’s capability to reduce diagnostic errors and optimize resource utilization, thereby lowering overall healthcare costs (58–60). However, our findings also reveal methodological limitations in current health economic evaluations (HEEs), such as inadequate reporting of key metrics like Incremental Cost-Effectiveness Ratios. This observation resonates with previous studies highlighting the need for rigorous reporting standards to ensure reliable assessments of AI interventions’ cost-effectiveness (61–63). Addressing these methodological shortcomings is crucial for accurately determining AI’s economic value in healthcare settings While economic models, such as those described by Abramoff et al. (56) and Khanna et al. (53), forecast considerable cost savings, these projections are based on idealized scenarios. While these evaluations underscore substantial potential savings associated with AI implementation, it is important to note that most current economics are predominately based on simulated or idealized scenarios.

Real-world implementation often reveals complexities—such as integration costs, infrastructural limitations, and clinician resistance—that may significantly moderate anticipated economic benefits (13). Therefore, robust, real-world pilot studies, comprehensive sensitivity analyses, and clear reporting of Incremental Cost-Effectiveness Ratios (ICERs) are imperative to accurately determine the true economic viability of AI technologies in diverse healthcare contexts. Many economic analyses rest on “perfect-world” assumptions—omitting the hidden costs and operational complexities that arise during real-life deployment. For example, theoretical cost-saving models rarely account for (1) prolonged IT integration and customization with legacy electronic health record systems; (2) dedicated clinician and support-staff training time and associated productivity losses; (3) workflow redesign efforts, user acceptance testing, and iterative troubleshooting; (4) fluctuating reimbursement schemes tied to performance metrics or seasonal case volumes; and (5) variations in local labor markets, infrastructure readiness, and scale-dependent pricing. These unmodeled factors can substantially erode projected savings and may even reverse anticipated financial gains.

The ethical challenges identified in our review, including biases in AI algorithms and concerns about patient autonomy, are well-documented in existing literature. Nonetheless, we emphasize that current mitigation strategies, while promising, may not fully resolve these ethical challenges, and ongoing disparities could persist if oversight remains inadequate. For instance, studies have highlighted the potential of AI to perpetuate existing healthcare inequities if not properly managed (64–67). Our review also emphasizes the evolving global regulatory landscape, echoing analyses that stress the importance of harmonizing standards to manage data security and accountability effectively. We acknowledge that the path to global regulatory harmonization is complex and may be slower than anticipated due to varying regional priorities and legal frameworks. Additionally, our focus on the balance between regulatory oversight and technological innovation aligns with discussions on the need to foster innovation while ensuring safety and efficacy. Navigating these ethical and regulatory challenges is essential to prevent exacerbating health disparities and to promote equitable healthcare delivery. Although frameworks like the FAIR statement are promising steps advances in addressing and tackling ethical and equity concerns, we recognize that these guidelines are still developing and have not yet fully resolved deeply entrenched ethical challenges, such as dataset bias, algorithmic fairness, and patient autonomy. Frameworks such as FAIR and HEAL provide initial guidance for bias mitigation, yet empirical evidence for their effectiveness is limited. The HEAL case study reported high equity metrics for certain racial and sex categories but observed performance gaps in non-cancer conditions. Implementation of fairness frameworks therefore requires continuous auditing across diverse populations.

Continuous empirical validation across diverse populations remains essential, alongside iterative refinements informed by real-world clinical experiences, to ensure that AI-driven healthcare advances do not inadvertently exacerbate existing disparities.

Our review highlights the necessity of structured governance frameworks and proactive risk management strategies for AI integration in clinical practice. We emphasize that while proposed governance models offer promising guidelines, their implementation remains challenging and requires tailored approaches in different healthcare contexts. This is consistent with studies advocating for comprehensive governance models to ensure the safe deployment of AI technologies (68–70).

Further, AI’s integration into health policy represents an essential advancement in public health strategy and crisis management. Ramezani et al. (57) illustrated AI’s vital role in health policy decision-making processes, particularly through real-time analytics, geospatial intelligence, and predictive modeling. Such AI-driven methodologies proved highly effective during public health crises, enhancing policy responsiveness, targeted resource allocation, and proactive risk mitigation strategies.

The identification of implementation barriers, such as data interoperability challenges and trust deficits, aligns with existing literature emphasizing the need for multidisciplinary collaboration to address these issues effectively (71, 72). Furthermore, our findings suggest that continuous system validation and adaptation are crucial to maintain AI performance in real-world settings. Developing robust governance structures and fostering collaboration among stakeholders are pivotal for the successful and sustainable implementation of AI in healthcare.

Our review underscores the economic promise of AI in healthcare, particularly in enhancing therapeutic interventions and optimizing clinical workflows. These findings resonate with broader industry analyses. Accenture, for example, estimated that AI applications could save the US healthcare economy up to $150 billion annually by 2026 through efficiencies in areas like robot-assisted surgery and virtual nursing assistants (73). However, the high initial investments required for AI implementation present challenges, especially for underfunded healthcare systems. Kastrup et al. (2024) emphasized the necessity of comprehensive cost–benefit analyses to ensure that AI technologies deliver value proportionate to their costs (43).

It is crucial to align the detailed methodological caution presented in the narrative with the consistent data summaries provided in the tables and appendices. Although the tables provide a structured overview of study characteristics and quality assessments, they do not fully capture the nuanced uncertainties inherent in many of the included studies. Readers should therefore interpret quantitative summaries through the lens of the narrative’s focus on the potential overestimation of benefits and the limitations of simulated models. This combined perspective underscores the importance of applying these findings cautiously and with context-awareness in real-world settings.

### Integrated adaptive AI translation framework (IA^2^TF)

We developed the Integrated Adaptive AI Translation Framework (IA^2^TF) as a conceptual model pending real-world implementation and validation. It synthesizes recurring themes from high-quality studies: data interoperability and standardization repeatedly identified as implementation barriers; continuous monitoring and retraining emphasized in prospective validation; ethical oversight and fairness highlighted in bias-mitigation reviews; variation in regulatory frameworks underscoring the need for hybrid governance; and multidisciplinary collaboration consistently described as a facilitator of adoption. IA^2^TF integrates these insights into five interrelated components—data interoperability, real-world performance monitoring, ethical oversight, hybrid regulatory governance, and interdisciplinary collaboration—and, as a theoretical proposal, requires prospective evaluation.

### Data interoperability and standardization

Lack of seamless data exchange and common standards is a well-recognized barrier to AI integration in healthcare. The IA^2^TF prioritizes robust data interoperability by advocating for universally adopted data standards and interoperable health information infrastructure. In practice, this means integrating protocols such as HL7 FHIR, DICOM, and other open standards into AI development and deployment processes to ensure that algorithms can access and interpret diverse clinical data sources (74). By standardizing data formats, ontologies, and quality controls across institutions, the framework mitigates the silos and inconsistencies that often hinder the applicability of AI across multiple sites.

Standardization of data collection and curation not only improves model generalizability but also facilitates external validation, since models trained on diverse, standardized datasets are more likely to perform reliably in new settings (74). By ensuring data are “fair, accessible, interoperable, and reusable” in the AI context, this component directly addresses the current lack of interoperability and lays a strong foundation for scalable AI deployment. In sum, the IA^2^TF’s emphasis on data interoperability rectifies a fundamental translational hurdle, enabling AI systems to leverage comprehensive, high-quality data streams from real-world practice and thus better reflect clinical reality.

### Real-world performance monitoring and adaptive learning

A critical limitation in current AI implementations is the drop-off in performance when models move from controlled trials to complex real-world environments. The Real-World Performance Monitoring and Adaptive Learning component of IA^2^TF establishes a continuous post-deployment monitoring regime to track AI systems’ clinical performance over time. This draws on the principle that AI model performance can evolve or degrade due to changes in data input or practice patterns, necessitating ongoing vigilance (75). Under our framework, health institutions would implement systematic performance audits—evaluating metrics like accuracy, error rates, and patient outcomes *in situ*—to detect performance drift or emergent safety issues. Crucially, IA^2^TF couples monitoring with an adaptive learning mechanism: when performance deviates beyond pre-defined thresholds, processes are in place to recalibrate or retrain the model on new data (with appropriate oversight) or even to temporarily suspend the AI tool if needed (75). This dynamic life-cycle approach aligns with regulators’ recent emphasis on AI “total product life cycle” management, wherein “acceptable performance at validation does not guarantee sustained adequacy” and that timely interventions trough retraining or model updates is essential for maintaining safety (75). By capturing real-world efficacy data—including actual clinical outcomes and resource impacts—this component also moves beyond hypothetical economic projections, providing empirical evidence of cost-effectiveness and clinical value in everyday practice. In effect, IA^2^TF transforms AI implementation into a learning system: continuous feedback loops identify shortcomings, inform iterative improvements, and thereby maintain a high level of performance and reliability in the face of changing real-world conditions (75). This not only addresses the problem of poor real-world model performance but also builds a reservoir of real-world evidence that can refine future AI development and bolster stakeholder confidence in AI tools over time.

### Ethical oversight and transparent reporting

Our review underscored how algorithmic bias, opaque “black-box” models, and threats to patient autonomy remain prevalent challenges, underscoring the need for vigorous ethical safeguards. The IA^2^TF introduces a formal ethical oversight structure to be embedded at every stage of the AI deployment process. This entails establishing interdisciplinary ethics committees or review boards that evaluate AI systems for fairness, equity, and respect for patient rights before and during clinical integration. Proactive measures include bias audits on algorithms (assessing performance across demographic subgroups), requiring diverse and representative training data, and upholding patient privacy protections beyond basic regulatory requirements. Concurrently, the framework demands transparent reporting of AI model intentions, decisions, and limitations. In practice, this requires that for any AI tool utilized in clinical settings, its developers or deploying institution must clearly document and communicate the model’s purpose, data provenance, accuracy (including confidence intervals), and known failure modes or biases.

Such transparency can be operationalized through publicly available model factsheets or “model cards,” as well as adherence to reporting guidelines for AI in healthcare studies (for example, CONSORT-AI extensions for clinical trials) (13). By demanding transparency, the IA^2^TF ensures that developers and users accountable for an AI system’s behavior and outcomes, aligning with global calls to place “ethics and human rights at the heart of AI design, deployment, and use” (28). This component uniquely tackles the trust deficit often faced by AI: when clinicians and patients can understand an algorithm’s role and see that it is under active ethical supervision, they are more likely to trust and appropriately use the tool. Moreover, rigorous ethical governance ensures that AI interventions do not inadvertently exacerbate health disparities—a risk highlighted by multiple studies—but instead actively contribute to fairness and patient autonomy. In summary, IA^2^TF’s ethical oversight and transparency mandates promote accountability, mitigate bias, and foster trust, thereby strengthening the moral and social legitimacy of AI in healthcare.

### Hybrid regulatory and governance models

Given the fragmented and evolving regulatory landscape for AI in healthcare—where different regions apply varying standards and traditional medical device regulations lag AI’s iterative nature—the IA^2^TF suggests a hybrid regulatory and governance model. This model harmonizes external regulation with internal governance to ensure both compliance and agility. Externally, the framework promotes alignment with international best practices in AI oversight. Such as utilizing aspects of the EU’s Medical Device Regulation and the FDA’s Software-as-a-Medical-Device (SaMD) guidelines as reference points for baseline safety and efficacy requirements. It also encourages global regulators to collaborate on shared principles (via forums similar to the International Medical Device Regulators Forum) to reduce discrepancies and facilitate global deployment while maintaining rigorous standards.

However, recognizing that no single regulatory approval guarantees long-term safety in all contexts, the IA^2^TF emphasizes robust local governance within healthcare organizations. We propose that each implementing institution establish an AI governance committee (or integrate into existing clinical governance structures) that includes diverse expertise—clinicians, data scientists, ethicists, and importantly legal/regulatory advisors (76). These committees are responsible for supervising AI tools throughout their life cycle within the organization, spanning from pre-deployment assessment to post-market monitoring, in coordination with the Real-World Performance component.

The “hybrid” nature of this model lies is characterized by its two-way communication: local governance bodies can rapidly address context-specific issues (such as a hospital identifying a workflow problem or safety concern with an AI tool and intervene immediately), while also feeding real-world performance data and observed challenges back to national regulators and manufacturers.

In this model, regulators model would allow adaptive updates to AI systems, such as model retraining or software improvements, under supervised conditions, rather than requiring entirely new approvals, thereby facilitating safe innovation.

This cooperative approach has been advocated in emerging policy discussions—for example, the FDA and other agencies emphasize ongoing monitoring but leave specifics to implementers, indicating the need for structured institutional governance. By combining top-down regulatory standards with bottom-up governance and feedback, the IA^2^TF’s hybrid model ensures that AI systems remain compliant with safety norms while continuously evolving. It addresses the current regulatory fragmentation by creating linked networks of oversight rather than isolated silos. Ultimately, this model accelerates the translation of AI into practice by providing a clear yet flexible oversight pathway: one that protects patients and quality of care without stifling technological adaptation or cross-border innovation.

### Interdisciplinary collaboration and continuous improvement

The final pillar of IA^2^TF is a commitment to interdisciplinary collaboration and a culture of continuous improvement, which is essential given the multifaceted nature of AI implementation. Successful translation of AI from bench to bedside cannot occur in a vacuum; it requires the active involvement of multiple stakeholders. Our framework formalizes mechanisms for collaboration across different domains of expertise. During AI system design and validation, clinicians, data scientists, engineers, ethicists, and health system administrators are brought together to ensure the tool addresses a real clinical need, is user-friendly, and adheres to ethical norms (13). This co-development approach has proven effective in enhancing usability and acceptance, as end-users are more inclined to trust and adopt AI solutions they helped design (13). During the deployment and implementation stage, the framework emphasizes comprehensive training and education for healthcare professionals, ensuring they comprehend the AI’s functioning and limitations—an effort that builds AI literacy and reduces resistance stemming from misunderstanding (13). The IA^2^TF further promotes engaging patients and the public in the AI process. This can for example be achieved through patient advisory panels or public consultations for new AI services, aiming to improve transparency and support a patient-centered design approach.

Importantly, interdisciplinary collaboration within IA^2^TF is ongoing and integrated into continuous improvement cycles. Multidisciplinary teams regularly review performance data alongside ethical and operational feedback from the field (gathered through the monitoring component) and collectively brainstorm and implement improvements or remedial measures.

For example, if the adaptive monitoring system identifies a decline in an AI tool’s accuracy within a particular patient subgroup, data scientists and clinicians will jointly investigate the issue, adjust the model or workflow, and evaluate the outcomes of that intervention.

Likewise, if frontline staff report workflow hurdles or patient concerns, the governance committee and technical teams coordinate to refine protocols or user interfaces. This iterative, collaborative problem-solving embodies a learning health system ethos, wherein the technology and its use protocols are continuously refined to enhance safety, effectiveness, and user satisfaction. By breaking traditional silos, the IA^2^TF ensures that expertise is shared across disciplines—for instance, that legal experts inform developers about regulatory boundaries, or that clinicians inform data scientists about nuances of patient care that a model might miss. This collaboration speeds up problem-solving and innovation, leading to AI systems that are more suited to the clinical environment and capable of evolving over time.

Ultimately, this component addresses the often fragmented and single-discipline approach to AI deployment by instilling collective ownership and accountability. It advances the field by demonstrating how continuous interdisciplinary engagement leads to safer, more effective AI implementations that can scale across different settings and populations. In essence, human governance and learning are placed on equal footing with technological advancement, ensuring that AI in healthcare remains a participatory and evolving enterprise, rather than a static product launch.

Advancing the Field: The IA^2^TF represents a novel and integrative approach that directly addresses the underlying causes behind the translational gap in healthcare AI.

By simultaneously fortifying data foundations, ensuring real-world validation, maintaining ethical integrity, innovating oversight models, and bringing together diverse stakeholders, this framework offers a comprehensive solution rather than fragmented remedies.

It operationalizes many of the abstract recommendations from prior studies and guidelines, such as the implementation of standardized evaluations, ethical oversight, and regulatory harmonization, which experts have identified as prerequisites for sustainable AI integration.

Methodologically, IA^2^TF advances the field toward higher rigor and relevance. By encouraging prospective real-world studies, continuous model updates, and transparent reporting, future AI tools will be supported by stronger evidence and subject to ongoing scrutiny, thereby narrowing the evidence gap between efficacy (performance under ideal conditions) and effectiveness (performance in practical scenarios).

Ethically, the framework embeds fairness and accountability into the AI life cycle, moving the needle from reactive risk management to proactive ethical design and oversight. Operationally, the IA^2^TF updates governance by combining the strengths of regulatory standards with the agility of local control and the innovation of interdisciplinary teams. This approach fosters an ecosystem where AI systems are scalable (through interoperable design and stakeholder engagement), trustworthy (through ethical practices and transparency), and continuously clinically validated (through adaptive monitoring and evidence generation).

This integrated adaptive approach is set to bridge the enduring gap between AI development and its tangible effects on clinical practice.

By addressing the multifaceted barriers in unity, the IA^2^TF can accelerate the translation of AI innovations into routine, safe, and equitable patient care, thereby advancing the field of healthcare AI from isolated successes to sustainable, system-wide transformation.

The next section summarizes the framework components, inputs, responsible parties, and outputs in a tabular format for clarity and as a quick reference guide for implementation (Table 3).

**Table 3:** IA²TF components, inputs, stakeholders, and outputs.

Framework component	Key inputs	Responsible stakeholders	Primary outputs
Co-design & problem definition (Pillar 1)	Clinical problem statement Patient needs & context Data availability & quality assessment Ethical guidelines/principles	Clinicians, Patients/Advocates, Data Scientists, Ethicists, Health Administrators	Clearly defined use-case and AI solution concept Requirements specification (clinical and technical) Ethical and equity considerations documented (e.g. FAIR principles applied)
Model development & validation (Pillar 2)	Curated training and validation datasets Algorithm selection and design criteria Design specifications from Pillar 1 Regulatory guidelines/standards for AI (if applicable)	Data Scientists/Developers, Clinician Researchers, Biostatisticians, (Regulatory advisors)	Trained AI model (prototype and subsequent refined versions) Validation results (performance metrics, bias analysis) Model documentation (including intended use and limitations) Regulatory submission dossier (if seeking approval)
Clinical integration & deployment (Pillar 3)	Validated AI model ready for use Health IT infrastructure (EHR integration, APIs) User training materials and support Regulatory approval/clearance (if required)	Hospital IT Specialists, Clinicians (end-users), Health Administrators, Implementation Specialists, (Regulators for compliance)	Deployed AI system within clinical workflow Trained users (completion of training sessions) Operational protocols and usage guidelines (SOPs) Initial deployment evaluation report (pilot results and user feedback)
Continuous monitoring & evaluation (Pillar 4)	Real-world usage data (model outputs and corresponding clinical outcomes) Performance metrics definitions and thresholds User feedback reports and incident logs Equity monitoring plan (outcome stratifiers by group)	Quality & Safety Team, Data Analysts, Clinicians (reporting), Patients (feedback), AI Governance Committee	Periodic performance reports (accuracy, errors, outcome impact) Bias and fairness audit results (if any disparities found) Logged incidents and analysis of root causes Alerts or trigger reports for any metric exceeding risk thresholds
Adaptive learning & governance (Pillar 5)	Identified model issues or performance gaps (from Pillar 4) New data for model retraining or updates Ethical review inputs (if concerns raised) - Regulatory change protocols (pre-specified change plans)	AI Governance Board (multidisciplinary), Data Scientists, Ethicists, Clinician Leaders, Regulators (for oversight on major changes)	Decisions on action: e.g. approval of updating model or modify usage policy Updated AI model version or adjusted algorithm parameters Re-validation reports for updated model (test results, safety checks) Revised deployment (model redeployed or usage guidelines updated) Communication of changes to stakeholders (including users and regulators)

The table provides a summary of each component (pillar or phase) of the Integrated Adaptive AI Translation Framework, along with its key inputs, responsible stakeholders, and primary outputs.

This structured summary serves as a checklist for implementing each part of the framework, ensuring clarity of roles and deliverables.

**Figure 2:**
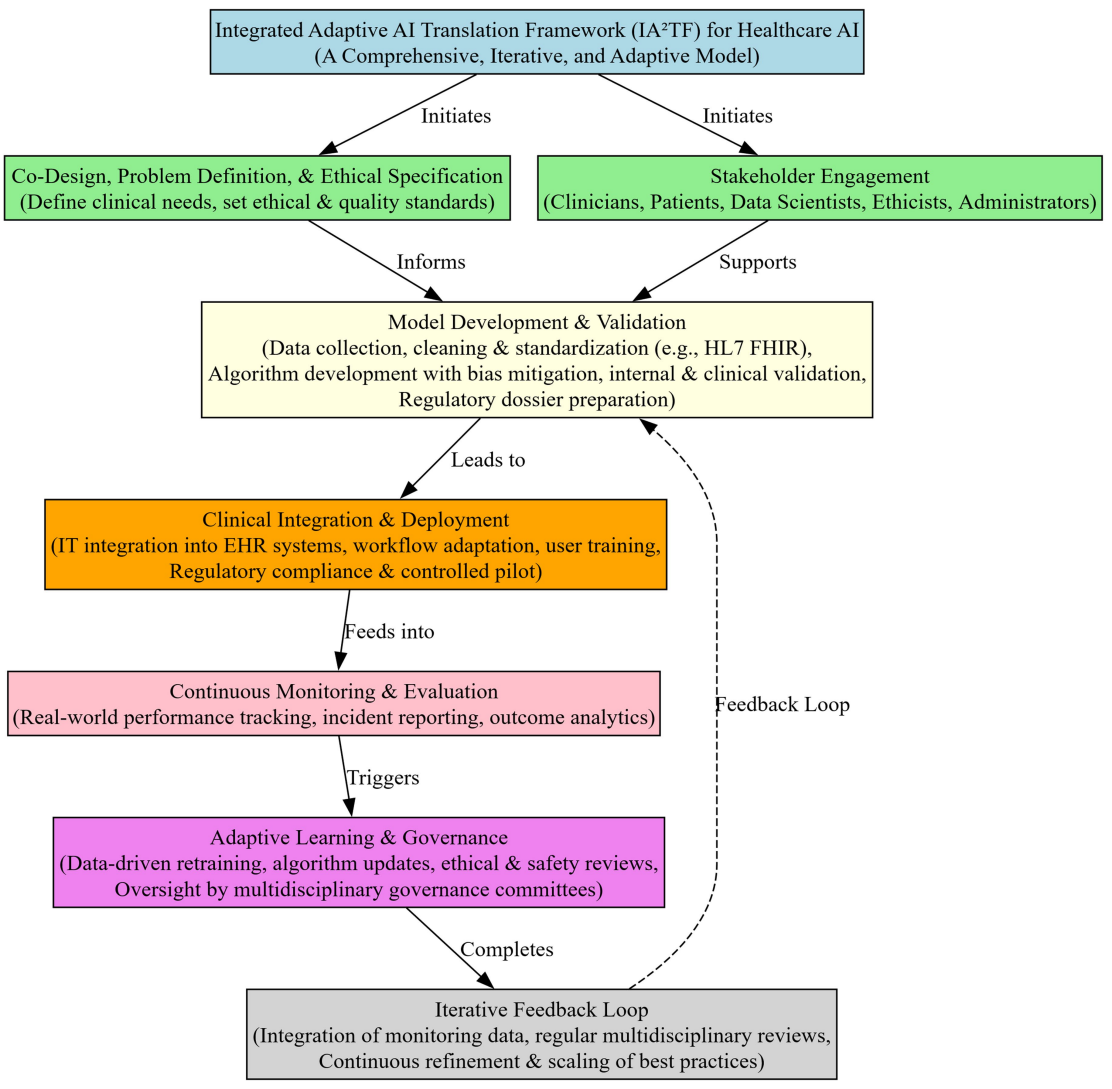
Integrated adaptive AI translation framework (IA^2^TF).

By following the IA^2^TF and using tools like the above table, healthcare organizations and AI developers can systematically translate AI innovations into clinical reality. The framework’s integrated and adaptive nature positions it as a novel contribution to the AI-in-healthcare literature—one that balances innovation with caution, and automation with human oversight, to ultimately achieve safe, effective, and continuously improving AI-supported healthcare (Figure 2).

### Recommendations and implications

The findings of our study, in combination with the novel Integrated Adaptive AI Translation Framework (IA^2^TF), underscore the urgent need to reimagine the integration of artificial intelligence in healthcare through comprehensive and forward-thinking strategies. Our recommendations advocate for the adoption of standardized, transparent methodologies for economic evaluations tailored specifically to AI. This approach involves rigorous pilot studies in diverse clinical settings to validate cost-effectiveness and scalability in real-world environments, ensuring that essential metrics—such as Incremental Cost-Effectiveness Ratios (ICERs)—are consistently reported and critically examined. Such robust evaluations, combined with innovative reimbursement models that merge the principles of Fee-for-Service and Value-Based Care, are crucial for establishing sustainable financing mechanisms that reflect the multifaceted economic benefits of AI technologies.

In parallel, it is imperative to embed stringent ethical standards into every facet of AI integration. Proactively addressing algorithmic biases and promoting equity requires AI systems to be trained on diverse and representative datasets, following globally recognized ethical guidelines like the FAIR statement. Ongoing ethical oversight, coupled with adaptive corrective actions, will protect patient autonomy and build the trust necessary for widespread acceptance of AI innovations in clinical practice.

Furthermore, the need for global regulatory harmonization is paramount considering the disparate legal landscapes currently governing healthcare technologies. Policymakers are urged to pursue incremental yet decisive steps toward unified regulatory frameworks, drawing on successful models like the European Union’s Medical Device Regulation and the FDA’s guidelines for Software as a Medical Device. Establishing public-private partnerships that bring together regulators, healthcare providers, industry leaders, and academic experts will accelerate the development of regulations that enhance patient safety, streamline compliance, and simultaneously nurture innovation.

Healthcare institutions must also prioritize the establishment of dedicated, multidisciplinary governance structures that are agile enough to oversee the entire lifecycle of AI integration. By forming governance committees composed of clinicians, data scientists, ethicists, legal experts, and administrators, organizations can implement proactive risk management strategies that include continuous performance monitoring, local retraining, and adaptive risk mapping. This proactive governance is vital for ensuring that AI systems remain safe, reliable, and effective over time, thereby reinforcing the trust of both clinicians and patients.

In addition to these institutional imperatives, the role of AI in shaping health policy and enhancing public health management cannot be overstated. Policymakers should harness AI-driven analytics and predictive modeling to inform evidence-based decisions and optimize resource allocation, particularly in times of crisis. Strategic investments in AI-powered surveillance systems, capable of real-time analysis, will significantly improve the responsiveness and effectiveness of public health initiatives.

Equally important is the emphasis on multidisciplinary collaboration and professional education. The successful integration of AI in healthcare is contingent upon the seamless cooperation of diverse stakeholders, from clinicians and data scientists to ethicists and policymakers. Strengthening these collaborations, alongside comprehensive initiatives to enhance AI literacy among healthcare professionals, will not only foster the development of ethically sound and clinically relevant AI solutions but also ensure their continuous improvement and rigorous evaluation.

The establishment of secure and comprehensive data governance frameworks is also fundamental to the long-term success of AI integration. By advocating for widely accepted healthcare data standards such as HL7 FHIR and DICOM, stakeholders can overcome the challenges of data interoperability, thereby enabling efficient data exchange that is essential for continuous AI training, validation, and improvement.

Finally, active patients and public engagement must be central to the AI development process. Transparent communication about the capabilities, limitations, and intended uses of AI systems will build informed trust and promote a patient-centered approach to innovation. Involving patients and public representatives throughout the lifecycle of AI integration ensures that technological advancements are aligned with actual healthcare needs and ethical imperatives.

Overall, these recommendations and implications provide a strategic blueprint for transforming the current AI landscape into healthcare. They call for a balanced integration of rigorous economic evaluations, ethical vigilance, regulatory harmonization, robust governance, interdisciplinary collaboration, and enhanced data infrastructure. Implementing these measures allows healthcare systems to develop AI innovations that are technologically advanced, ethically sound, economically viable, and seamlessly integrated into clinical practice, ultimately enhancing patient care globally.

### Strengths and limitations

This integrative review offers a comprehensive synthesis of diverse evidence regarding AI applications in healthcare, covering economic evaluations, ethical analyses, regulatory comparisons, and risk management frameworks. The use of a two-phase composite quality assessment—drawing on established tools such as PRISMA, AMSTAR 2, and the Drummond checklist—ensured that studies were critically appraised and weighted according to methodological rigor. In addition, the structured thematic synthesis across economic, ethical, and governance domains provides actionable recommendations for policymakers and healthcare stakeholders.

Despite its broad scope, the review is constrained by the heterogeneity of the included studies, with varying designs and inconsistent reporting standards. Many findings rely on hypothetical models and simulated scenarios, which may not fully capture the complexities of real-world implementation. The variable quality of the underlying evidence—ranging from rigorous systematic reviews to less robust narrative commentaries—necessitates cautious interpretation. Furthermore, discrepancies in study details and occasional inconsistencies in citation and table references highlight challenges in data standardization. These limitations underscore the need for more rigorous, prospective studies to validate model-based projections and ensure that policy recommendations are substantiated by robust empirical evidence.

## Conclusion

Artificial intelligence holds transformative potential for healthcare by enhancing diagnostic accuracy, operational efficiency, and cost-effectiveness. By synthesizing diverse evidence across economic, ethical, and governance domains, we reveal that AI innovations can significantly reduce expenditures and improve clinical workflows, provided that rigorous methodologies and transparent reporting are maintained. Nevertheless, challenges persist, including algorithmic bias, data inequities, and fragmented regulatory frameworks that compromise patient safety and autonomy. To overcome these obstacles, we advocate for standardized economic evaluations, robust ethical oversight, and globally harmonized regulatory standards. Moreover, effective risk management and adaptive governance are critical to bridge the gap between controlled research environments and real-world application. Multidisciplinary collaboration, active clinician engagement, and ongoing pilot studies will refine AI implementation, ultimately ensuring sustainable, equitable healthcare delivery and improved patient outcomes across diverse settings, yielding measurable, lasting improvements. To address these challenges, we proposed the Integrated Adaptive AI Translation Framework (IA^2^TF), a comprehensive roadmap that emphasizes data standardization, real-world performance monitoring, ethical oversight, hybrid regulatory models, and interdisciplinary collaboration.

## Data Availability

The original contributions presented in the study are included in the article/Supplementary material, further inquiries can be directed to the corresponding author.

## Glossary

AI: Artificial Intelligence
AMSTAR 2: A MeaSurement Tool to Assess systematic Reviews 2
API(s): Application Programming Interface(s)
CFIR: Consolidated Framework for Implementation Research
DICOM: Digital Imaging and Communications in Medicine
DL: Deep Learning
EHR: Electronic Health Record
ERM: Enterprise Risk Management
FAIR: Findable, Accessible, Interoperable, and Reusable
FDA: Food and Drug Administration
FFS: Fee-for-Service
FMECA: Failure Mode, Effects, and Criticality Analysis
GDPR: General Data Protection Regulation
HEDIS: Healthcare Effectiveness Data and Information Set
HEAL: (Framework for) Health Equity Assessment and/or Learning
HL7 FHIR: Health Level Seven – Fast Healthcare Interoperability Resources
IA2TF: Integrated Adaptive AI Translation Framework
ICER(s): Incremental Cost-Effectiveness Ratio(s)
LLMs: Large Language Models
ML: Machine Learning
MIPS: Merit-based Incentive Payment System
PRISM: Preferred Reporting Items for Systematic Reviews and Meta-Analyses
RAF: (CMS) Risk Adjustment Factor
RCTs: Randomized Cons
SaMD: Software as a Medical Device
SOP(s): Standard Operating Procedure(s)
VBC: Value-Based Care
VinDr-CXR: VinDr Chest X-Ray
WHO: World Health Organization
