# Supplementary material for: Economic, ethical, and regulatory dimensions of artificial intelligence in healthcare: an integrative review
Source: Front Public Health. 2025 Aug 29;13:1617138. doi: 10.3389/fpubh.2025.1617138 (PMC12425912; doi:10.3389/fpubh.2025.1617138)
Supplement: Supplementary file 1 [file Table_1.docx]

Appendix 1: Study characteristics of included studies

| Study (Authors, Year) | Study Design/Methodology | Objectives/Aims | Population/Setting | Key Methods/Frameworks | Main Findings/Results | Recommendations/Implications | Limitations/Special Considerations |
| --- | --- | --- | --- | --- | --- | --- | --- |
| Ueda et al. (2024) | Review Article (Invited Review) | Examine fairness issues in AI healthcare (radiology focus) | Healthcare AI in radiology | Comprehensive literature review; focus on data, algorithmic, & interaction biases; FAIR recommendations | Defined fairness; identified multiple bias sources; proposed mitigation strategies | Adopt diverse datasets, regular audits, quality control, & education | Literature‐based; generalizability may be limited |
| Ramezani et al. (2023) | Scoping Review | Explore AI applications in health policy using the policy triangle framework | Global health policy contexts | Policy triangle (context, content, actors, process); qualitative synthesis | AI enhances situational awareness, communication, & stakeholder engagement; aids agenda setting & evaluation | Leverage AI for proactive policymaking & improved public health interventions | Broad scope; variability in study quality and context |
| Abramoff et al. (2024) | Policy Review Article | Analyze reimbursement models (FFS vs. VBC) for medical AI adoption | Healthcare reimbursement systems; diabetic eye exam case study | Comparative policy analysis; review of reimbursement strategies | FFS offers clear processes; VBC aligns with outcomes; hybrid approaches may be optimal | Pursue dual/hybrid reimbursement models to scale AI adoption | Administrative delays; challenges for smaller startups |
| Nguyen et al. (2022) | Prospective Validation Study | Evaluate the real-world performance of the VinDr-CXR AI system | Phu Tho General Hospital, Vietnam (6,285 chest X-rays) | Prospective validation; comparison with radiologist reports; performance metrics (F1, accuracy, sensitivity, specificity) | Clinical performance (F1 = 0.653) was lower than lab results (F1 = 0.831); moderate accuracy, sensitivity, & specificity observed | Continuous retraining and enhanced human-AI collaboration are needed | Distribution shifts; data matching challenges; underrepresentation of specific conditions |
| Liao et al. (2022) | Perspective (Case Study) | Describe the development of a governance framework for clinical AI | University of Wisconsin Health system | Multi-domain governance model (clinical, operational, leadership); oversight committees | Improved clinician trust, patient safety, & successful deployment of multiple AI applications | Implement structured, multi-domain governance with continuous monitoring | Case-specific context; potential limitations in generalizability |
| Khanna et al. (2022) | Systematic Review (Economic Analysis) | Compare economic impacts of AI in diagnosis vs. treatment | Healthcare systems; economic modeling settings | Economic modeling; hypothetical cohort analysis; cost-effectiveness evaluation | AI reduces costs; treatment applications yield greater economic benefits; projected cumulative savings over 10 years | Leverage dual reimbursement strategies with focus on treatment benefits | Based on hypothetical models; real-world variability may differ |
| Ferrara et al. (2024) | Systematic Review | Assess the impact of AI on clinical risk management & patient safety | Clinical settings (diagnostics, medication, incident reporting) | Literature review organized by ICPS taxonomy | AI improves diagnostic accuracy, reduces medication errors, & streamlines incident reporting; potential for false alerts noted | Maintain human oversight alongside AI integration in risk management | AI may introduce cognitive overload; continuous validation is required |
| Chomutare et al. (2022) | Scoping Review | Identify barriers & facilitators for AI implementation via the CFIR framework | Diverse clinical settings | Application of the CFIR framework; systematic review of implementation studies | Facilitators include leadership, stakeholder engagement, & robust validation; barriers include interoperability, data quality, & trust issues | Enhance interdisciplinary collaboration and address technical barriers | Implementation challenges may be context-specific |
| Di Palma et al. (2025) | Research Article (ERM Framework) | Propose an Enterprise Risk Management (ERM) framework for AI-related risks | Healthcare institutions integrating AI applications | ERM framework using FMECA, RCA, HAZOP; systematic risk mapping & monitoring | Comprehensive risk identification across clinical, technical, ethical, & financial domains demonstrated | Promote interdisciplinary risk management, continuous monitoring, & improved transparency | Framework requires significant organizational change; broader validation needed |
| Mennella et al. (2024) | Narrative Review | Explore ethical, regulatory, & legal challenges of AI in healthcare | General healthcare settings | Comprehensive review of ethical principles & regulatory frameworks | Identified challenges in patient autonomy, equity, transparency, & accountability | Establish robust governance frameworks with clear accountability mechanisms | Narrative synthesis; may lack systematic quantification |
| Schaekermann et al. (2024) | Quantitative Case Study (Framework Validation) | Introduce and validate the HEAL framework for equity assessment in AI performance | Dermatology AI model; data from USA & Australia | Four-step equity assessment; "top-3 agreement" metric | High equity metrics for racial/ethnic groups & females; variable performance across age groups; equity gaps in non-cancer conditions | Refine the framework and improve AI performance for underrepresented groups; further intersectional analysis recommended | Limited demographic representation; further research required |
| Pesapane et al. (2021) | Narrative Review | Examine evolving legal & regulatory frameworks for healthcare AI post-pandemic | Global (EU, US, China, Russia) | Comparative narrative review; analysis of regulatory guidelines (e.g., MDR, GDPR) | Accelerated regulatory changes; challenges in data privacy, accountability, & harmonization noted | Develop flexible and harmonized legal frameworks to support safe AI integration | National variability and rapidly evolving regulations pose challenges |
| Wang & Zhang (2024) | Narrative Review | Review applications & challenges of large language models (LLMs) in healthcare | Medical & healthcare sectors (clinical and research applications) | Literature review of LLM applications in medical Q&A, EHR generation, & imaging analysis | LLMs show promise across domains; face challenges in data security, accuracy, fairness, & explainability | Ensure continuous model validation, improved data management, & enhanced transparency measures | Rapidly evolving technology; ethical and accuracy challenges remain |
| Li et al. (2024) | Narrative Review | Explore AI’s potential & challenges in personalized healthcare | Personalized care settings (patients & providers) | Comprehensive analysis of applications (chatbots, remote monitoring, predictive analytics) | AI enhances patient engagement, treatment personalization, & remote monitoring; integration challenges noted | Foster interdisciplinary collaboration, robust policy development, & regulatory oversight | Data security, bias, & regulatory hurdles remain significant challenges |
| Moro-Visconti et al. (2023) | Narrative Review & Sensitivity Analysis (with Network Theory) | Assess AI-driven scalability and its impact on firm sustainability & valuation | Traditional firms across various industries | Sensitivity analysis, network theory, & economic simulations | AI adoption improves EBITDA, revenue, & firm valuation; sensitivity analysis shows potential doubling/tripling of equity | Leverage AI for operational efficiency while managing cybersecurity and bias risks | Economic models may oversimplify complexities; regulatory & ethical challenges persist |
| Kastrup et al. (2024) | Systematic Review (Methodological Quality) | Evaluate the methodological quality of health economic evaluations of AI | Economic evaluations of AI interventions | Systematic review; quality assessment of ICER reporting & resource use documentation | Significant gaps in methodological rigor; lack of AI-specific guidelines; poor reporting of cost-effectiveness metrics | Develop comprehensive AI-specific methodological frameworks and guidelines | Limited number of high-quality evaluations; design variability exists |
| Darwiesh et al. (2023) | Research Article (Quantitative Analysis & Case Study) | Propose and validate an AI model for risk management using social media data | Healthcare institutions; CVS Health case study (USA) | NLP and big data analytics; risk identification, assessment, & monitoring; quantitative performance metrics | Achieved ~81% overall accuracy; detailed categorization of operational, financial, & other risks | Enhance model accuracy, reduce computational complexity, & validate externally across institutions | Challenges with data complexity, social media noise, & external validity |

**Note: Studies are categorized by design. Narrative reviews, while valuable for theoretical insights, do not employ systematic risk-of-bias methods and should be interpreted with caution relative to systematic reviews and empirical studies which provide more rigorous methodological assessments.**

**Abbreviation List**

- **AI:** Artificial Intelligence
- **CFIR:** Consolidated Framework for Implementation Research
- **CVS:** CVS Health (healthcare company)
- **EHR:** Electronic Health Record
- **ERM:** Enterprise Risk Management
- **FAIR:** Findable, Accessible, Interoperable, Reusable
- **FFS:** Fee-for-Service
- **F1:** F1 Score (harmonic mean of precision and recall)
- **FMECA:** Failure Modes, Effects, and Criticality Analysis
- **GDPR:** General Data Protection Regulation
- **HAZOP:** Hazard and Operability Analysis/Study
- **HEAL:** Health Equity Assessment in AI (framework)
- **ICER:** Incremental Cost-Effectiveness Ratio
- **ICPS:** International Classification for Patient Safety
- **LLM(s):** Large Language Model(s)
- **MDR:** Medical Device Regulation
- **NLP:** Natural Language Processing
- **RCA:** Root Cause Analysis
- **USA:** United States of America
- **VBC:** Value-Based Care
- **EBITDA:** Earnings Before Interest, Taxes, Depreciation, and Amortization
