# Supplementary material for: Economic, ethical, and regulatory dimensions of artificial intelligence in healthcare: an integrative review
Source: Front Public Health. 2025 Aug 29;13:1617138. doi: 10.3389/fpubh.2025.1617138 (PMC12425912; doi:10.3389/fpubh.2025.1617138)
Supplement: Supplementary file 2 [file Table_2.docx]

The following table represents the consolidated composite quality assessment of all included studies, combining evaluations of methodological rigor and reporting clarity.

Appendix 2: Composite quality assessment

| Authors et al | Article Type | Key Appraisal Criteria | Evaluation Summary |
| --- | --- | --- | --- |
| Ueda et al., 2023 | Narrative Review | Clarity of objectives; comprehensive literature coverage; transparency in limitations | Provides a clear discussion on fairness issues; lacks a structured risk‐of‐bias assessment. |
| Ramezani et al., 2023 | Scoping Review | Clear research question; transparent search strategy; systematic mapping of evidence | Demonstrates systematic mapping of the literature; limited by less rigorous appraisal of included studies. |
| Abramoff et al., 2024 | Policy Perspective/Commentary | Argument clarity; integration of case examples; discussion of stakeholder impact | Well-argued with relevant data; methodological details are limited due to its commentary format. |
| Nguyen et al., 2022 | Empirical Study | Robust study design; clear performance metrics (accuracy, F1 score); external validity | Methodologically sound with clear performance reporting; highlights challenges in translating lab findings to clinical practice. |
| Liao et al., 2022 | Perspective/Case Study | Multi-disciplinary oversight; practical outcomes; transparency in governance structure | Effectively outlines a governance model with early successes; underreports some limitations inherent to case studies. |
| Khanna et al., 2022 | Empirical Economic Analysis | Economic modeling; comparative analysis; cost-effectiveness metrics | Provides rigorous economic analysis with robust modeling and transparent discussion of assumptions. |
| Ferrara et al., 2024 | Systematic Review | Use of PRISMA guidelines; risk-of-bias assessment; comprehensive data synthesis | Exhibits high methodological rigor with a comprehensive search and critical appraisal process. |
| Chomutare et al., 2022 | Scoping Review | Integration of theoretical frameworks (e.g., CFIR); methodological transparency; systematic mapping of barriers/facilitators | Demonstrates clear mapping of implementation challenges and facilitators; theoretical grounding is strong despite variable reporting quality. |
| Di Palma et al., 2025 | Research Article/Case Study | Clear enterprise risk management (ERM) framework; practical case examples; discussion of challenges including “black box” issues | Innovatively integrates ERM with AI risk assessment; well-documented with actionable insights for healthcare risk managers. |
| Mennella et al., 2024 | Narrative Review | Identification of ethical/regulatory issues; comprehensive literature integration; critical reflection | Provides a thorough overview of ethical and regulatory challenges; lacks formal systematic appraisal methods seen in other studies. |
| Schaekermann et al., 2024 | Empirical Framework/Case Study | Framework development; robust quantitative analysis; equity focus | Rigorously develops the HEAL metric using robust statistical methods, effectively assessing performance equity across demographic groups. |
| Pesapane et al., 2021 | Review | Comparative analysis across jurisdictions; regulatory clarity; timeliness (post-pandemic focus) | Provides a thorough cross-national analysis; effectively addresses emerging regulatory challenges in the post-pandemic context. |
| Wang and Zhang, 2024 | Narrative/Semi-Systematic Review | Comprehensive literature coverage; balanced synthesis of advances and challenges; methodological transparency | Offers an extensive overview of LLM applications; clearly delineates challenges such as data security and bias, though inclusion criteria are variably applied. |
| Li et al., 2024 | Narrative Review | Detailed innovation description; discussion of technical/regulatory challenges; balanced perspective | Articulates innovative applications and associated challenges effectively; practical recommendations provided despite a lack of formal bias assessment. |
| Moro‑Visconti et al., 2023 | Empirical/Conceptual Study | Robust economic analysis; integration of network theory; sensitivity analysis | Employs advanced economic modeling and network theory to assess scalability; sensitivity analyses support the findings regarding financial and sustainability impacts. |
| Kastrup et al., 2024 | Systematic Review | Systematic methodology (PRISMA); data transparency; critical appraisal using established checklists | Methodologically robust review that highlights quality gaps in current economic evaluations and suggests specific areas for improvement. |
| Darwiesh et al., 2023 | Empirical Study/Model Proposal | Model clarity; innovative use of NLP and social media data; mathematical formulation; practical case study validation | Presents a well-defined risk management model with strong methodological innovation; includes explicit mathematical formulations and a case study that validates its practical utility. |
